# Supplementary material for: Green Oxidation of Amines by a Novel Cold-Adapted Monoamine Oxidase MAO P3 from Psychrophilic Fungi Pseudogymnoascus sp. P3
Source: Molecules. 2021 Oct 15;26(20):6237. doi: 10.3390/molecules26206237 (PMC8538783; doi:10.3390/molecules26206237)
Supplement: Supplementary file 1 [file molecules-26-06237-s001.zip › molecules-1421359-supplementary.pdf]

Green oxidation of amines by a novel cold-adapted monoamine oxidase MAO P3 from psychrophilic fungi *Pseudogymnoascus* sp. P3

Iga Jodłowska, Aleksandra Twarda-Clapa, Kamil Szymczak, Aneta M. Białkowska

SUPPLEMENTARY INFORMATION

Contents:

Figure S1. Growth of fungi on induction medium……………………………………………………………………1

Strain Certificate S1. Characteristics of psychrophilic *Pseudogymnoascus* sp. P3 strain……………………………2

Method S1. Phylogenetic trees……………………………………………………………………………………3

Method S2. Hydrogen peroxide strip test…………………………………………………………………………4

Method S3. Determination of K_m and V_{max} values based on Lineweaver-Burk curves……………………………5

Method S4. GC-MS spectra………………………………………………………………………………………6

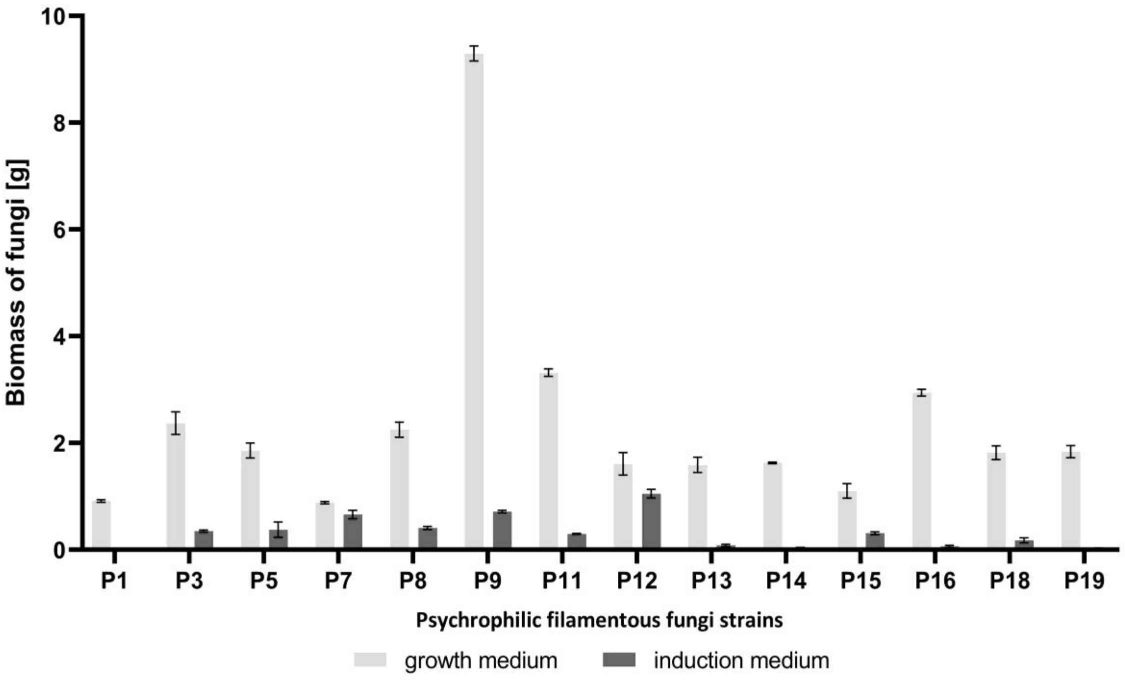

Figure S1. Growth of fungi on induction medium containing 0.1% of n-butylamine as a nitrogen source. Biomass of fungi was collected by filtration from 125 m**L** of culture, the presented data represent wet mass.

Strain Certificate S1. Characteristics of psychrophilic Pseudogymnoascus sp. P3 strain

Group of microorganisms: microscopic fungi **Date of isolation:** 2002

The origin of the strain: Hills above the Arctowski Station overgrown with bryophytes and lichens – samples taken from the soil surface

1. Macroscopic characteristic of fungal strain

growth: on the surface and submerged
size: 15 – 35 mm
shape: irregular
edges: flocculus
color: white
surroundings : undyed (yellow only within the border of colonies)
structure: irregular

Macroscopic images of the strain (solid and liquid culture)

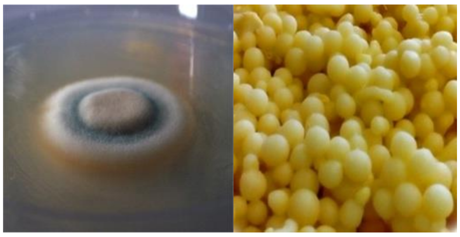

Microscopic image of the strain (40x)

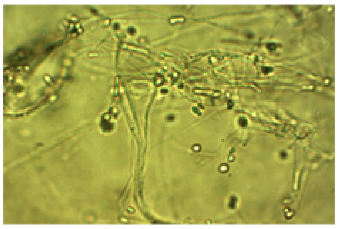

2. Morphological features

- **Microscopic description:** while grown at 10˚C, the produced conidia had shape from sphere to globoid.

3. Features of growth/culture

- **Growth characteristics on different media:**
- ✓ **Czapek – solid**: single colonies;
- ✓ **Czapek – liquid**: in stationary conditions growth on the medium surface, in shaking conditions in shapes of small balls

4. Physiological features

- ✓ **Productions of dyes on solid medium**: yellow dye, does not diffuse outside the mycelium.
- ✓ **Production of dyes in liquid medium**: yellow dye, in the entire volume of the medium.

5. Influence of temperature on growth of fungi (solid medium)

2°C	6°C	10°C	15°C	20°C	30°C	37°C
+	+	++	+	+	-	-

"+++"- strong growth; "++"-good growth; "+"-weak growth; "-"-no growth

Optimal temperature : 10°C

6. Range of tolerance for NaCl

1% - 10%

7. Biochemical characteristic

Production of enzymes:

- ✓ β - galactosidase
- ✓ Xylanase
- ✓ Cellulase
- ✓ Pectinase
- ✓ Amylase
- ✓ Pectin lyase
- ✓ Monoamine oxidase
- ✓ Keratinase
- ✓ Lipase
- ✓ Intracellular proteases
- ✓ Tannases

Degradation of different substrates on solid medium:

- ✓ degradation of starch
- ✓ degradation of tributyrin
- ✓ degradation of gelatin
- ✓ degradation of casein from milk

8. Production of fatty acids

- ✓ Mitric acid (C14:0)
- ✓ Palmitic acid (C16:0)
- ✓ Palitoleic acid (C16:1 Δ7)
- ✓ Stearic acid (C18:0)
- ✓ Oleic acid (C18:1 Δ9)
- ✓ Linoleic acid (C18:2 Δ9,12)
- ✓ α-linoleic acid (ALA, C18:3 Δ9,12,15)

9. Ability to grow and produce enzymes on mediums containing waste from the food industry

Ability to grow on wastes from food industry:

Waste type	Growth
Oranges skins	+++
Pumpkin skin homogenate	+++
Brewing spent grain	++
Watermelon skin homogenate	+++
Corn cob residues	+++
Apple pomace	+++

"+++"- strong growth; "++"-good growth; "+"-weak growth; "-"-no growth

Production of enzymes on medium containing wastes from the food industry:

Waste type	cellulase	xylanase	pectinase
Oranges skins	-	++	+++
Pumpkin skin homogenate	+	-	+++
Brewing spent grain	-	+	++
Watermelon skin homogenate	+	++	+++
Corn cob residues	+	+	++
Apple pomace	-	-	++

"+++"- strong activity; "++"-good activity; "+"-weak activity; "-"-no activity.

Method S1. Phylogenetic trees

Multiple sequence alignments were performed using MSA tool MUSCLE @EBI (https://www.ebi.ac.uk/Tools/msa/muscle/) for sequences of ITS1/5.8s rRNA/ITS2 region of P3 strain compared to several *Pseudogymnoascus* species, as well as several psychrophilic or mesophilic fungi from *Ascomycota* division. Phylogenetic trees were created in MEGA X software (www.megasoftware.net) with the use of three different statistical methods of phylogenetic tree generation (Maximum Likelihood Tree, UPGMA Tree, Maximum Parsimony Tree) and Bootstrap set to 500. Accession numbers are visible next to the species names.

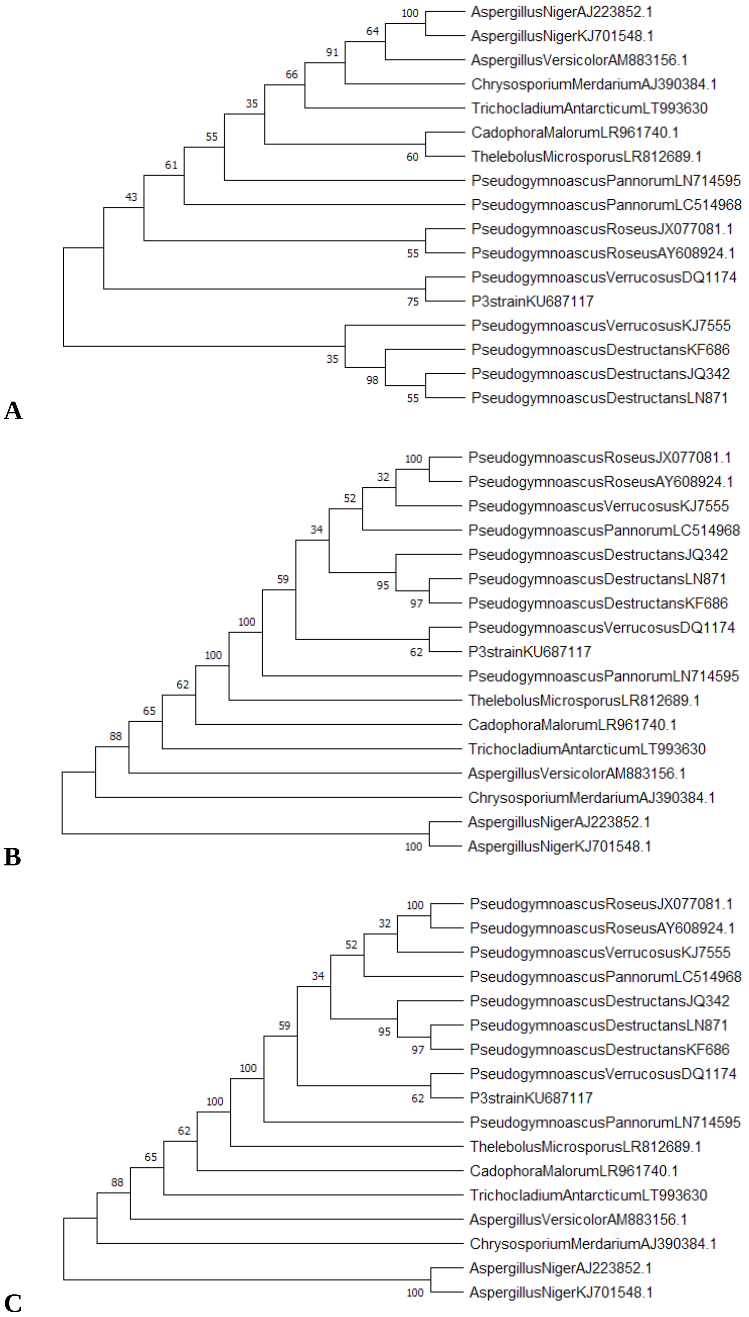

Figure S2. Phylogenetic trees based on analysis of ITS1/5.8s rRNA/ITS2 regions. MUSCLE tool was used for MSA. Bootstrap consensus trees shown for three different statistical methods of tree generation: A. Maximum Likelihood Tree B. UPGMA Tree C. Maximum Parsimony Tree.

Method S2. Hydrogen peroxide strip test

During reaction of oxidative deamination of amines catalyzed by MAO, one of the side product is H_2O_2, which can be detected by H_2O_2 strip test. Test was performed after reaction of oxidative deamination of primary (*n*-butylamine) and secondary (6,6-dimethyl-3-azabicyclohexane) amines. Total volume of the reaction was 1 mL, where was 0.5 mL of protein extract, 0.25 mL of 0.01 M of potassium phosphate buffer pH 7.2 and 0.25 mL of 20 mM amine in 0.01 M of potassium phosphate buffer pH 7.2. Reaction was performed for 1 h, in 30˚C, 810 rpm. After specified time, the amount of produced H_2O_2 was checked by strip test, where the intensity of blue color is a measurement of the side product concentration.

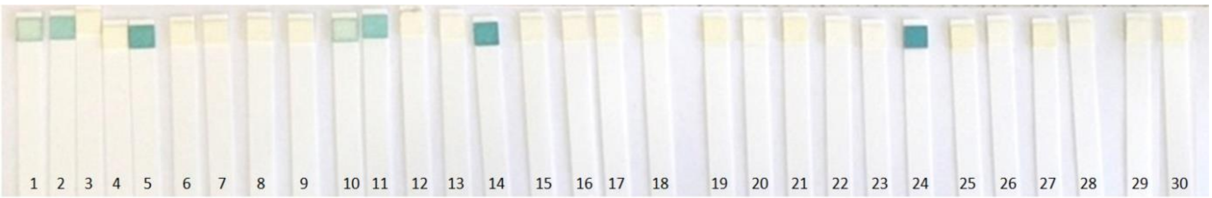

Figure S3. Result of hydrogen peroxide strip test. Qualitative method for identification of potential producer of amine oxidase. 1- P3 1st amine; 2- P3 2nd amine; 3- P1 1st amine; 4- P1 2nd amine; 5- P7 1st amine; 6- P7 2nd amine; 7- P8 1st amine; 8- P8 2nd amine; 9- control 1st amine; 10- P5 1st amine; 11- P5 2nd amine; 12- P9 1st amine; 13-P9 2nd amine; 14- P15 1st amine; 15- P15 2nd amine; 16- P16 1st amine; 17- P16 2nd amine; 18- P14 1st amine; 19- P14 2nd amine; 20- P12 1st amine; 21- P12 2nd amine; 22- P14 1st amine; 23- P14 2nd amine; 24- P11 1st amine; 25- P11 2nd amine; 26- P18 1st amine; 27- P18 2nd amine; 28- control 2nd amine; 29- P19 1st amine; 30- P19 2nd amine. The intensity of blue color represent the presence of hydrogen peroxide in reaction mixture.

Production of H_2O_2 was observed for P3, P5 strains in reaction with primary (*n*-butylamine) and secondary (6,6-dimethyl-3-azabicyclohexane) amines, and for P7, P15, P11 strains in reaction with primary (*n*-butylamine) amine. Only for strain P19 we were able to observe synthesis of H_2O_2 during reaction with secondary (6,6-dimethyl-3-azabicyclohexane) amine.

Method S3. Determination of K_m and V_{max} values based on Lineweaver-Burk curves

The double-reciprocal curve was create based on the inverse initial velocity as a function of the inverse substrate concentration. Curves were created for each amine substrate (Fig. S2), based on the results of GC-MS analysis (Paragraph 2.7).

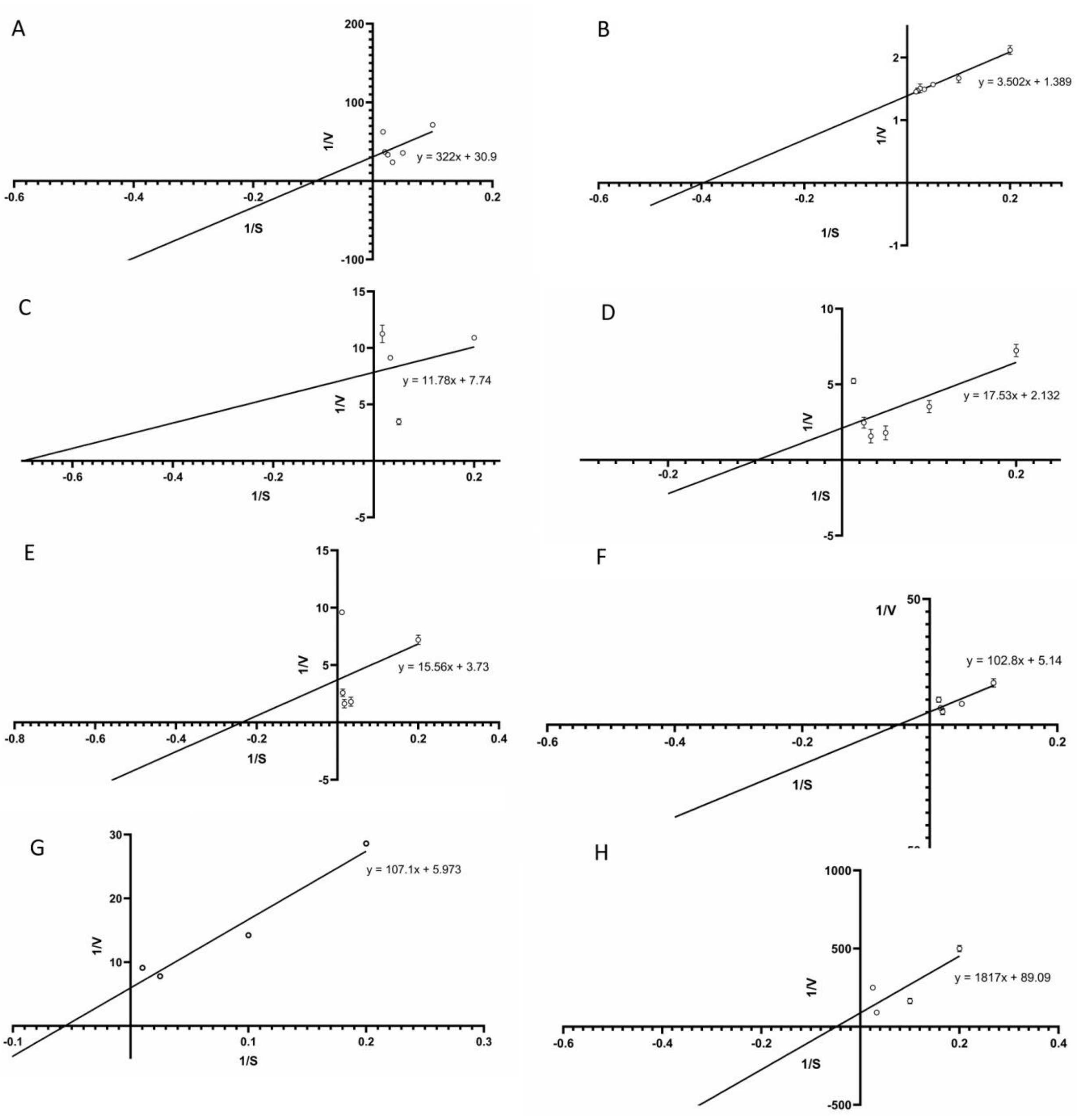

Figure S4. The double-reciprocal curves for MAO P3 amines substrates: A. 3-azabicyclo[3.3.0]octane; B. hexamethyleneimine; C. 6,6-dimethyl-3-azabicyclohexane; D. cyclopentylamine; E. α-methylbenzylamine; F. pyrrolidine; G. *sec*-butylamine; H. indoline.

Method S4. GC-MS results

We tested MAO P3 ability to oxidize various amine substrates (Paragraph 3.9-3.10). Psychrophilic MAO was able to oxidize 8 substrates out of 16 tested. In supplementary information's, we show the GC-MS result for each substrate.

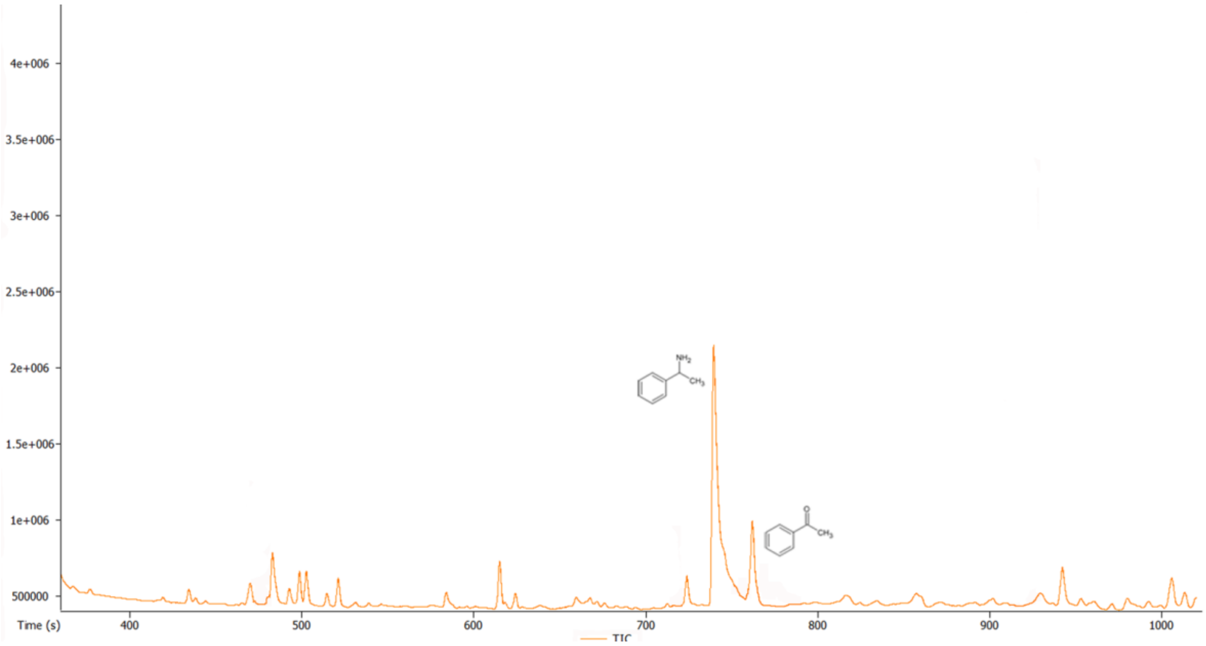

Figure S5. GC-MS analysis result of oxidative deamination of α-methylbenzylamine to acetophenone. α-methylbenzylamine retention time 12.3 min (m/z peaks 44;79;106); Acetophenone retention time 12.7 min (m/z peaks 51;77;105)

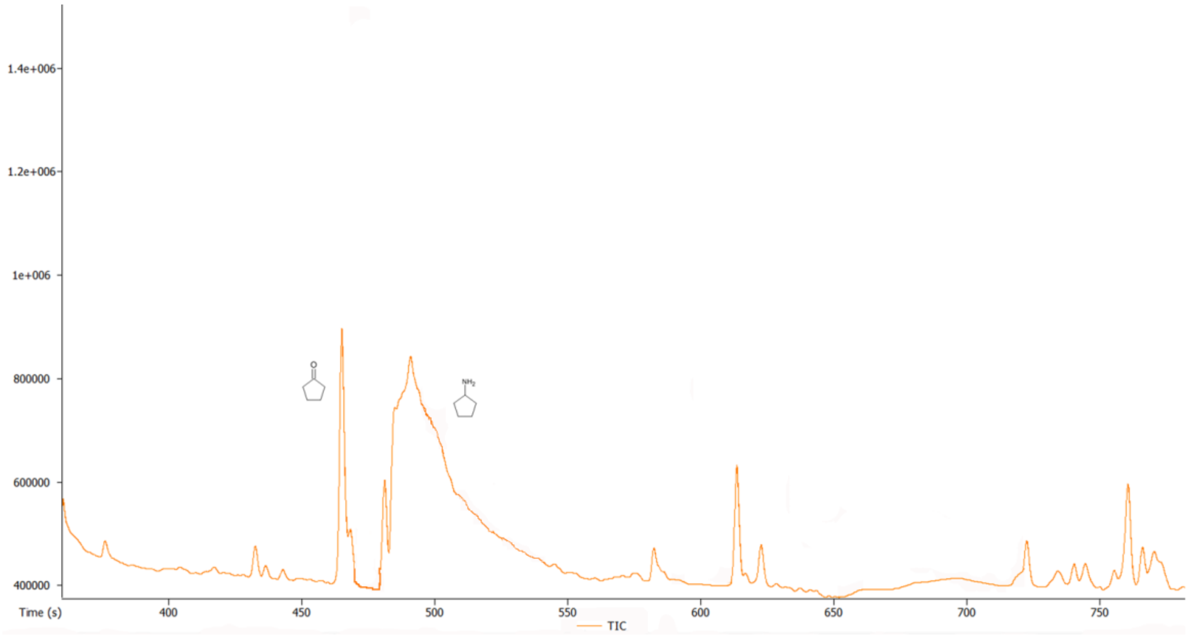

Figure S6. GC-MS analysis of oxidative deamination of cyclopentylamine to cyclopentanone. Cyclopentylamine retention time 8.2 min (m/z peaks 56;85); cyclopentanone retention time 7.75 min (m/z peaks 55;84)

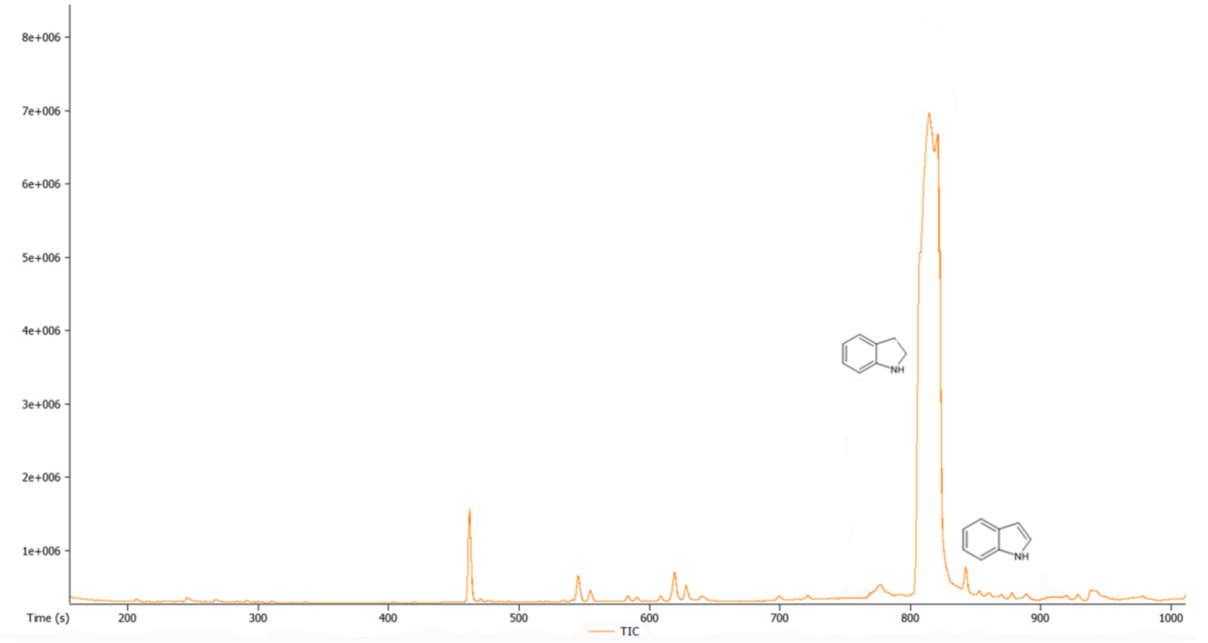

Figure S7. GC-MS analysis of oxidative deamination of indoline to imine - indole. Indoline retention time 13.43 min (m/z peaks 63;91;118), Indole retention time 13.68 min (m/z peaks 63;90;117).

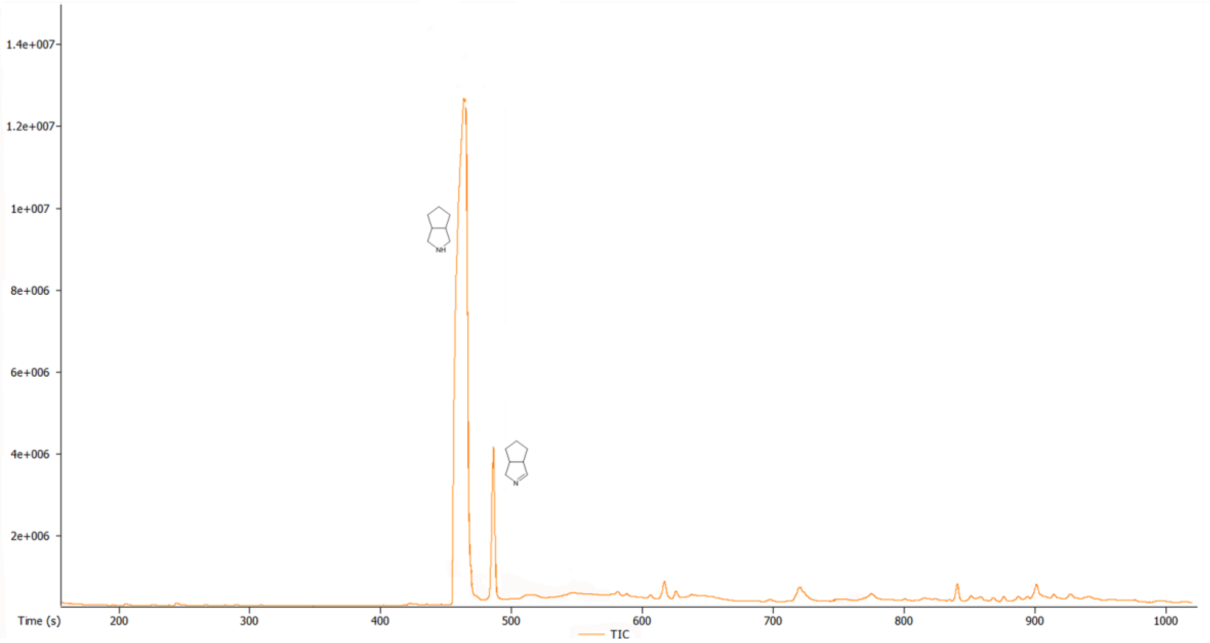

Figure S8. GC-MS analysis of oxidative deamination of 3-azabicyclo[3.3.0]octane to imine – 3-azabicyclo[3.3.0]oct-2-ene. 3-azabicyclo[3.3.0]octane retention time 7.59 min (m/z peaks 43;68;111); 3-azabicyclo[3.3.0]oct-2-en retention time 8.15 min (m/z peaks 43;68;109).

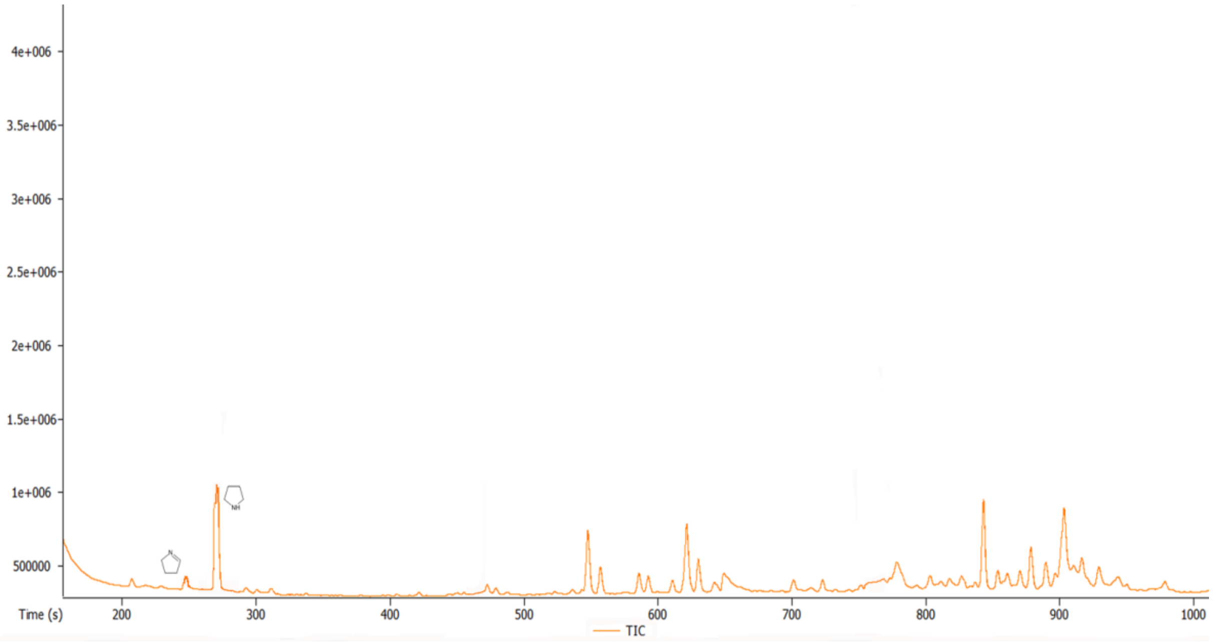

Figure S9. GC-MS analysis of oxidative deamination of pyrrolidine to imine 1-pyrroline. Pyrrolidine retention time 4.7 min (m/z peaks 43;70); 1-pyrroline retention time 4.2 min (m/z peaks 41;69).

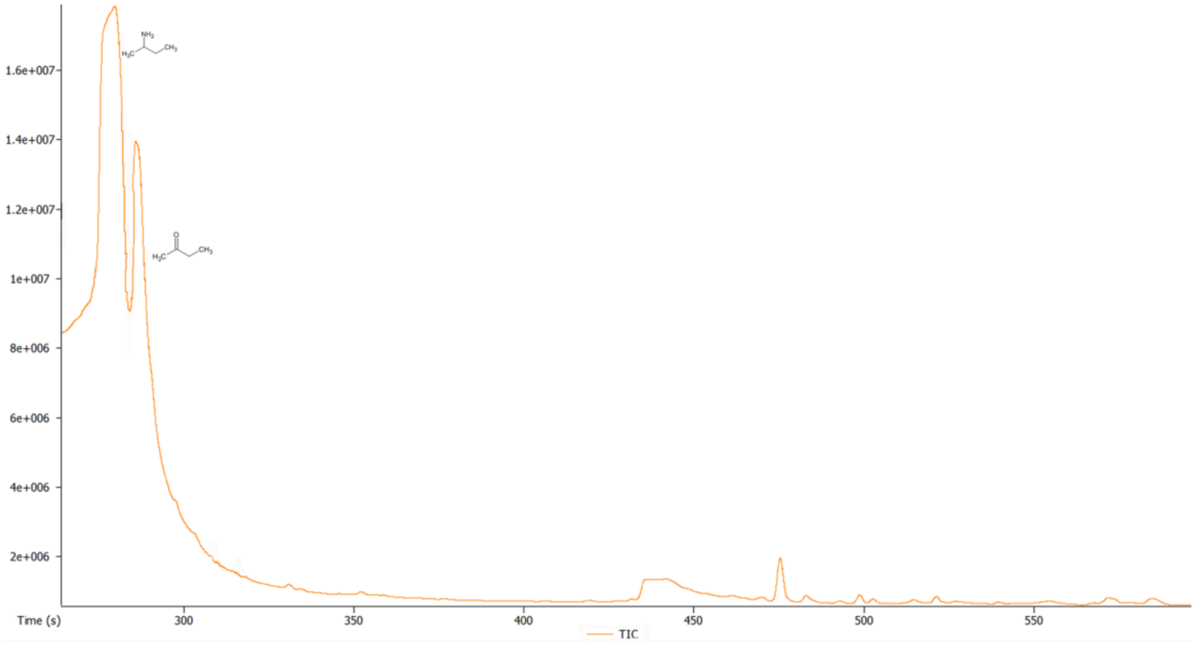

Figure S10. GC-MS analysis of oxidative deamination of *sec*-butylamine to 2-butanone. *sec*-butylamine retention time 4.5 min (m/z peaks 44;84); 2-butanone retention time 4.85 min (m/z peaks 43;84).

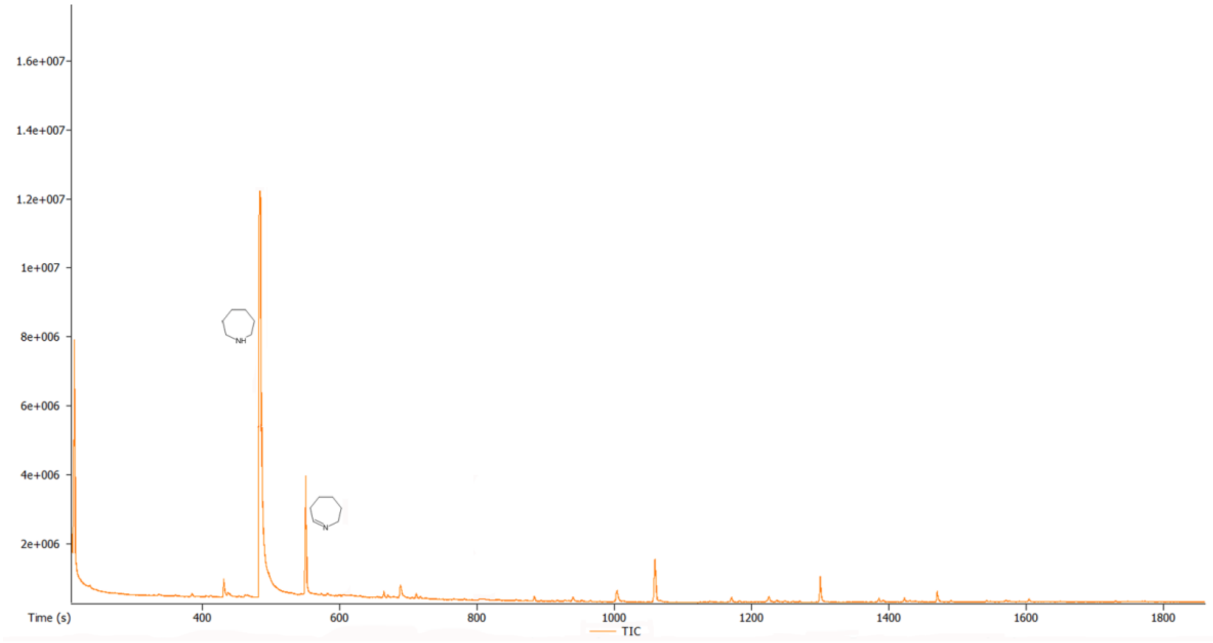

Figure S11. GC-MS analysis of oxidative deamination of hexamethyleneimine to 3,4,5,6-tetrahydro-2H-azepine. Hexamethyleneimine retention time 8.11 min (m/z peaks 30;43;70;99); 3,4,5,6-tetrahydro-2H-azepine retention time 9.33 min (m/z peaks 30;43;97).

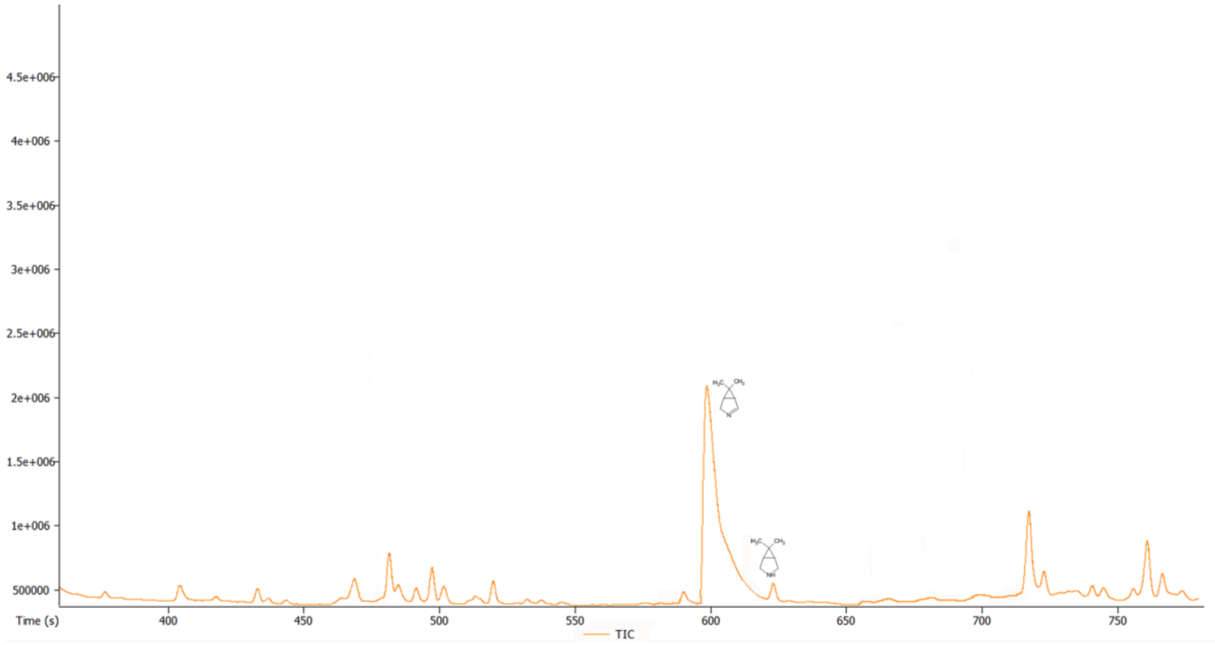

Figure S12. GC-MS analysis of oxidative deamination of 6,6-dimethylo-3-azabicyclo[3.1.0]hexane to 6,6-dimethylo-3-azabicyclo[3.1.0]hex-2-ene. 6,6-dimethylo-3-azabicyclo[3.1.0]hex-2-ene retention time 9.95 min(m/z peaks 67;109); 6,6-dimethylo-3-azabicyclo[3.1.0]hexane retention time 10.01 min(m/z peaks 67;111).
